# How to Evaluate the Accuracy of Symptom Checkers and Diagnostic Decision Support Systems: Symptom Checker Accuracy Reporting Framework (SCARF)

**DOI:** 10.2196/76168

**Published:** 2026-01-16

**Authors:** Marvin Kopka, Markus A Feufel

**Affiliations:** 1 Division of Ergonomics, Department of Psychology & Ergonomics (IPA), Technische Universität Berlin, Straße des 17. Juni 135, Berlin, 10623, Germany, 49 30-314-70806

**Keywords:** symptom checker, symptom assessment applications, evaluation, case vignettes, preclinical, decision support, large language model, data analysis, health technology assessment, impact evaluation, artificial intelligence, AI

## Abstract

Symptom checkers are apps and websites that assist medical laypeople in diagnosing their symptoms and determining which course of action to take. When evaluating these tools, previous studies primarily used an approach introduced a decade ago that lacked any type of quality control. Numerous studies have criticized this approach, and several empirical studies have sought to improve specific aspects of evaluations. However, even after a decade, a high-quality methodological framework for standardizing the evaluation of symptom checkers is still lacking. This paper synthesizes empirical studies to outline the Symptom Checker Accuracy Reporting Framework (SCARF) and a corresponding checklist for standardizing evaluations based on representative case selection, an externally and internally valid evaluation design, and metrics that increase cross-study comparability. This approach is supported by several open access resources to facilitate implementation. Ultimately, it should enhance the quality and comparability of future evaluations of online and artificial intelligence (AI)–based symptom checkers, diagnostic decision support systems, and large language models to enable meta-analyses and help stakeholders make more informed decisions.

## Introduction

Symptom checkers (also called “symptom assessment applications,” “online symptom checkers,” or “self-assessment applications”) are websites or mobile apps in which medical laypeople can enter their symptoms. The apps then provide potential diagnoses and “self-triage” advice. Self-triage advice refers to recommendations given in a precare setting to assist users in determining if, how urgently, and in which institution they should seek care. The first study to systematically analyze the accuracy of these apps was conducted in 2015, and their accuracy has been debated ever since [1]. This seminal study evaluated symptom checkers using 45 medical case vignettes (15 emergency care cases in which users would call the national emergency line or go directly to the emergency department, 15 nonemergency cases in which users would seek primary care, and 15 self-care cases in which users would treat symptoms themselves or wait to see if symptoms improve before seeking care) that were taken from various medical resources, including medical education textbooks. The gold standard solution—that is, the most appropriate action for each case—was determined by 2 physicians who independently rated each case and then discussed disagreements. An unrelated researcher entered all cases into the various symptom checkers, and the authors calculated the proportion of cases correctly solved as the main outcome. This procedure has been used in most subsequent studies, sometimes with slight modifications such as adding more vignettes and triage levels, using lay-friendly phrasing of the vignettes, or including large language models as symptom checkers [2-5]. However, most of these studies acknowledged limitations with this approach and called for improved methods. Systematic reviews that attempted to determine the accuracy of symptom checkers across multiple studies quickly reached the consensus that these methods were often of low quality and that cross-study comparability was limited [6-9]. In recent years, some studies have explicitly formalized this criticism, whereas others have proposed solutions to address it, including several of our own [6,10-14].

In this paper, we do not want to add to this criticism; instead, we present the Symptom Checker Accuracy Reporting Framework (SCARF) and a checklist that can (1) be used to conduct high-quality symptom checker evaluation studies and (2) standardize the evaluation procedure to increase cross-study comparability of symptom checkers and large language models. Because self-triage advice is arguably the most useful information for medical laypeople, this framework focuses on self-triage accuracy as the main outcome [15].

## Limitations and Challenges of Previous Methodologies

**Figure 1.**
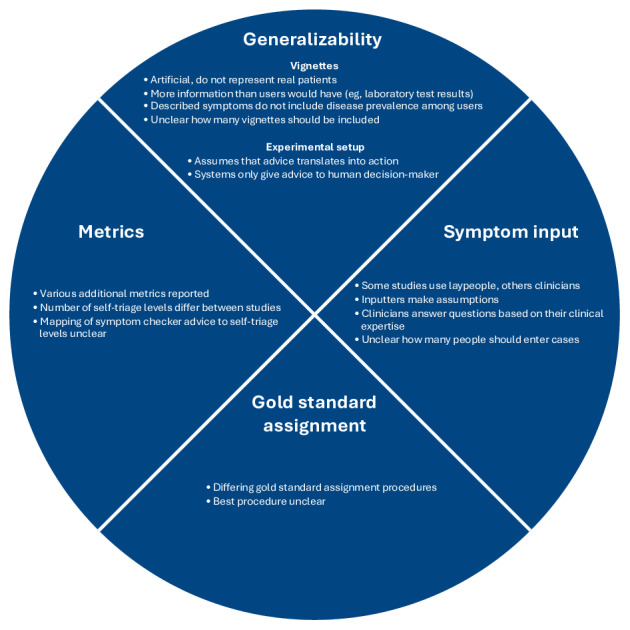
Four categories of criticism regarding symptom checker evaluation studies.

Most studies evaluating the triage accuracy of symptom checkers have criticized the existing evaluation approach for being artificial. In particular, the vignettes describe idealized, unambiguous cases, and some include scenarios for which symptom checkers would rarely be consulted (eg, recurrent aphthous stomatitis, which may be unexplainable upon first appearance but is easily recognized once experienced [16]). If the aim is to determine a triage accuracy metric that can be generalized to real-world interactions and scenarios in which symptom checkers are actually used, the inclusion of such cases in evaluations seems questionable. Apart from vignettes, current evaluation approaches have several other shortcomings that we grouped into 4 categories: generalizability, symptom input, gold standard assignment, and metrics (Figure 1). We build on these points to develop our standardized methodological evaluation framework.

The first point concerns the generalizability of the evaluations. This includes both the vignettes and the experimental setup, which, according to ecological validity theory, should resemble real-world use cases and interactions to yield results that can be generalized [17]. Traditional vignettes have been criticized for a lack of representativeness for several reasons. First, they are often derived from medical education textbooks and are therefore artificial, not representing the unspecific concerns for which patients would use a symptom checker [10,14]. Second, these cases are mostly written post hoc by clinicians who have access to more specialized information (eg, diagnoses, laboratory test results, and clinical examinations) than a patient consulting a symptom checker [10,13,18]. In other words, thus far, the information in existing vignettes does not reflect the types of problems actual users of symptom checkers face, and it is not clear what that information should be. Third, the cases described in the vignettes do not reflect the natural base rates of emergency or nonemergency versus self-care cases among users [10,13,14]. Fourth, there is no consensus on the number of vignettes that should be included in a vignette set or how to ensure their quality [10]. The experimental setup focuses on symptom checker accuracy and has thus been criticized for implicitly assuming that symptom checker advice directly translates into user actions, even though symptom checkers merely provide advice that users may or may not follow [19-21]. This limitation confines research to assessing only the “technical accuracy” of a symptom checker, without addressing its likely real-world impact. To determine whether technical accuracy translates into improved decision-making by users, symptom checkers ultimately need to be evaluated in user studies.

The second point concerns the procedure for inputting symptoms. Typically, a single person—who may or may not have medical expertise—enters the symptoms. Because not all information that a symptom checker might ask for is included in the vignette, the inputter must make assumptions about the case when asked about it. Thus, clinicians tend to rely on their clinical judgment and expertise, whereas laypeople—the actual users of symptom checkers—use various strategies, ranging from guessing to ignoring the questions they are asked [10,11,22]. It is also unclear how many inputters should be involved in the evaluation to yield valid performance estimates [10]. These issues suggest that the final output is highly dependent on the inputter or inputters, which creates an information bias that limits the internal validity of evaluation studies. This information bias is further compounded by the fact that different symptom checkers allow different input modalities (eg, free text, multiple-choice questions, images, or even laboratory results). These variations introduce an inherent comparability limitation, as the same case may be assessed differently depending on the input options of the tool.

The third point relates to the gold standard assignment used to assign the solution to a case vignette. Different studies use varying procedures: some use a single physician, others use multiple physicians, recordings from clinical encounters (such as telephone triage), or sometimes the authors even determine the gold standard solution themselves [10,12]. This variation not only limits the comparability between studies but also raises questions about the accuracy of the assigned gold standard in some cases.

The final point concerns the metrics used to evaluate symptom checkers. Although most studies report triage accuracy as the proportion of cases solved correctly, some also include additional metrics, such as the tendency to overtriage or undertriage and the safety of the advice [14]. The exact self-triage classifications differ between studies as well: for example, Semigran et al [1] used a 3-tiered approach including “emergencies,” “nonemergencies,” and “self-care cases,” whereas Hill et al [2] extended this classification to include “1-day urgent cases.” Furthermore, because different symptom checkers use varying classifications as well, it is unclear how their advice should be mapped to the study’s triage categories (eg, whether an urgent care clinic is considered emergency or nonemergency care). These issues ultimately limit cross-study comparability.

## Framework

**Figure 2.**
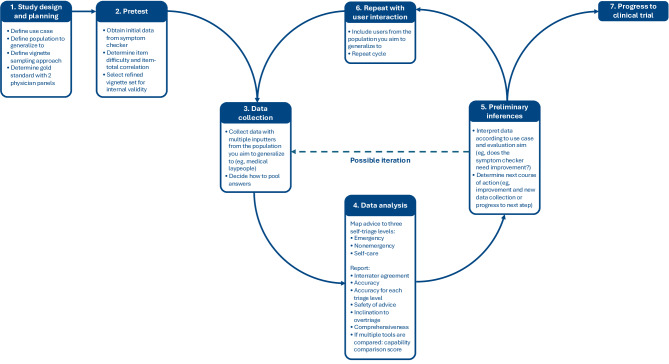
Integrated preclinical evaluation framework (Symptom Checker Accuracy Reporting Framework; SCARF) for evaluating the self-triage accuracy of symptom checkers and artificial intelligence–based tools.

To address these points, we developed an evaluation framework by integrating available empirical studies on methodological improvements. This framework can be found in Figure 2. It can be used for preclinical evaluations to identify symptom checkers that are likely to perform well in clinical trials and real-world evaluations. Once identified, the symptom checker should nonetheless undergo testing in a 3-phase clinical trial similar to pharmaceutical trials [23]. Hence, our framework not only standardizes vignette-based symptom checker evaluations but also makes subsequent clinical trials more cost efficient by identifying tools likely to yield positive outcomes. It can be applied both to evaluations across a broad set of cases as well as to those focusing on specific patient groups (eg, patients receiving rheumatology care), by defining the intended use case and population accordingly.

In the beginning (part 1), evaluators should clearly define the use case they intend to examine, such as “self-triage decisions” or “emergency care decisions.” Next, they should specify the target population to which they wish to generalize. For the self-triage use case, this might include symptom checker users deciding on their next course of action. Then, they should define a vignette sampling approach, which ensures that vignettes are representative of real patient cases and accurately reflect the disease or symptom and triage prevalence relevant to the use case. For example, the approach could sample real patient cases stratified according to the prevalence of symptom types entered into symptom checkers. A systematic sampling procedure to do that is available in the RepVig framework*,* and for the self-triage use case, a representative vignette set is provided in the framework’s validation study [13]. At this stage, researchers should also assign a gold standard solution to each case and define how the possible outputs of a symptom checker are mapped onto these categories. According to a study by El-Osta et al [12], this should involve 2 physician panels that independently rate the cases in focus groups and resolve any disagreement through discussion until consensus is reached.

Next, evaluators should obtain initial data from some symptom checkers to refine the vignette set according to test-theoretical criteria (part 2) to ensure that vignettes are not only externally but also internally valid. This process involves calculating the item-total correlation and excluding any cases with a negative or zero item-total correlation (to ensure that only cases accurately predicting overall performance are included). Additionally, item difficulty for each vignette should be determined, and cases with an item difficulty of zero may be excluded (to ensure that vignettes add meaningful information and are not impossible to solve). However, if these cases can be solved by physicians and are clinically plausible, even items with an item difficulty of zero may be retained in the vignette set to avoid inflating performance estimates. A procedure for this is outlined in one of our previous studies [24]. The size of the final vignette set should ultimately be determined using a power analysis. However, given that entering a large number of vignettes manually may be infeasible and that there is no empirical evidence on optimal set sizes, we pragmatically recommend a minimum of 45 vignettes. This number has proven feasible and has been applied across multiple studies [1,2,13,25], as it can be developed and entered by a single evaluator within a reasonable time frame, while still providing sufficient variation for a statistical analysis.

Using the refined vignette set, data from all symptom checkers can be collected (part 3). Multiple inputters (at least 2, possibly more) should enter every case into each symptom checker and select a “not sure” option in cases of missing information. To minimize inputter variability, inputters should follow a standardized protocol. For instance, Meczner et al [11] instructed inputters to enter only the symptoms explicitly stated in the vignette, allowing synonyms or broader categories but rejecting new information not included in the vignette. Their publication provides entry instructions that can be used in future studies to standardize input procedures. Once the inputters have obtained the data, their results should then be pooled. This can be achieved using several algorithms, but the best approach appears to be a majority vote, that is, the advice most frequently given to all evaluators [11]. For example, if 2 inputters receive the advice to seek emergency care while 1 inputter receives self-care advice, the recommendation should be coded as “emergency”.

In the next step, the data analysis (part 4), evaluators should map the received advice to a multitiered classification system. To increase comparability across studies and health care systems, we suggest using a 3-tiered classification system—“emergency,” “nonemergency,” and “self-care”—to provide a common reference structure. A potential “1-day-urgent” category could be classified as “nonemergency.” At the same time, we acknowledge that some systems use more granular triage categories; therefore, we suggest conducting sensitivity analyses (eg, treating “1-day-urgent” as “emergency” or as its own category, or analyzing the full set of available tiers) to assess the stability of the results. After mapping each recommendation, evaluators should first report the interrater reliability among all inputters to identify the influence of different inputters, followed by a set of metrics: overall accuracy, accuracy for each triage level, safety of advice, inclination to overtriage, and comprehensiveness [14,26]. These metrics were identified through systematic review of previously reported metrics and can increase comparability across different studies [14,26]. If multiple symptom checkers are evaluated simultaneously, we propose additionally reporting the Capability Comparison Score (developed in a previous study) to determine how symptom checkers perform relative to each other [14,26]. To assist researchers in reporting and visualizing these metrics, the R package *symptomcheckR* is available, where the formulas for calculating all metrics are described as well [26].

In the next step, the results should be interpreted according to the defined use case, and the next course of action should be determined (part 5): if developers aim to validate their tools, they may either decide to improve their tool and test it again (by going back to step 3) using the same setting or continue with the evaluation and test the best-performing tools with users in the loop making self-triage decisions (step 6). In this phase, users should be provided with the symptom checker, and the tool’s impact, instead of its “technical accuracy,” should be assessed in a new evaluation with sufficient statistical power [21]. This step is included because preclinical vignette studies can only benchmark technical accuracy and do not capture whether laypeople actually make better decisions when using a symptom checker. If results of user studies are also promising, the symptom checker can then be tested in a clinical trial with real patients and their symptoms to assess whether the symptom checker advice also translates to improved decisions by users in the real world (step 7).

## Open Questions

Our approach leaves several open questions for future research. First, some of the vignettes (such as the vignettes by Semigran et al [1] and our own [13]) do not include additional information for questions that symptom checkers may ask. Although some vignette sets do include additional information, there is no universal way to collect additional information. Future research could develop a method to supplement this missing information—perhaps using a hybrid approach that combines interviews with patients from whom the case vignettes were derived and synthetic artificial intelligence (AI)–generated supplementary data based on these interviews. Second, it remains unclear whether “accuracy” or a “correct” solution should be the main outcome. Perhaps a binary classification of correct versus incorrect in a task like symptom assessment that is associated with high uncertainty may be less relevant than assessing the impact of the advice—specifically, whether it is safe and appropriate for the individual and whether it increases or decreases health care demands. Third, with the introduction of large language models as an alternative to traditional symptom checkers, output variability plays an even greater role. Future research should address how to manage the variability of generated outputs when provided with identical inputs. Fourth, current evaluations do not specifically include atypical presentations. It remains unclear whether case vignettes are only suitable for typical cases or if vignette sets for atypical cases could also be developed. Although the RepVig framework could be used for developing such a vignette set again, assigning a reliable gold standard solution to atypical cases will be challenging [13]. Finally, our approach is highly tailored to a self-triage use case. Although it standardizes most aspects of an evaluation, diagnostic use cases may require additional details (such as clinical plausibility of the vignettes or a procedure to determine whether a diagnosis matches the gold standard) and outcome metrics (such as cumulative diagnostic scores [27]) that are not covered by our approach.

## Outlook

The SCARF (and the corresponding checklist in Multimedia Appendix 1 and Multimedia Appendix 2) presented in this paper addresses all previously raised points of criticism and aims to improve the quality and comparability of future symptom checker evaluations. However, we acknowledge that the presented approach is more resource intensive than the traditional approach introduced by Semigran et al [1] and may not be feasible for every evaluation. To aid researchers in integrating these methods into practice, several open resources are available for the presented use case: for example, representative vignettes are openly accessible and free to use [13], a refined vignette set that satisfies test-theoretical criteria is available as well [24], and all metrics can be easily calculated using the open-source symptomcheckR package [26]. We encourage researchers to build on these resources to improve the quality of future evaluations and enhance cross-study comparability.

## Conclusions

In this paper, we summarize the limitations and challenges of previous studies evaluating symptom checkers using vignettes. In recent years, several empirical studies have addressed most of these limitations individually, yet a unified methodological and reporting framework integrating these findings was missing. We present a preclinical framework and the corresponding SCARF checklist upon which future vignette-based symptom checker evaluations can build to address generalizability, input variability, gold standard assignment, and metrics, and we highlight several open access resources that evaluators can use. By adopting this approach, researchers can identify well-performing tools for more cost-efficient clinical trials and can significantly increase the quality and comparability of vignette-based symptom checker evaluation studies, thereby enabling reliable evidence syntheses. This can help move closer to assessing and improving the effectiveness of symptom checkers, diagnostic decision support systems, and large language models.

## Supplementary material

10.2196/76168 Multimedia Appendix 1 Symptom Checker Accuracy Reporting Framework (SCARF) checklist (editable version).

10.2196/76168 Multimedia Appendix 2 Symptom Checker Accuracy Reporting Framework (SCARF) checklist (PDF version).

## Abbreviations

AI: artificial intelligence
SCARF: symptom checker accuracy reporting framework
