# Supplementary material for: How to Evaluate the Accuracy of Symptom Checkers and Diagnostic Decision Support Systems: Symptom Checker Accuracy Reporting Framework (SCARF)
Source: JMIR Hum Factors. 2026 Jan 16;13:e76168. doi: 10.2196/76168 (PMC12810947; doi:10.2196/76168)
Supplement: Multimedia Appendix 1 [file humanfactors-v13-e76168-s001.docx]

**Symptom Checker Accuracy Reporting Framework (SCARF) Checklist**

| **Topic** | **Item Number** | **Item Description** | **Page Number** |
| --- | --- | --- | --- |
| **Title & Abstract** | | | |
| Title | 1 | Title should indicate that the study evaluates a symptom checker or diagnostic decision support system |  |
| Abstract | 2 | Summary of evaluation objective, methods, results, and conclusions |  |
| Introduction | | | |
| Background and Objectives | 3a | State the intended use case of the symptom checker (e.g., self-triage, emergency care triage) |  |
|  | 3b | Define the target population to which findings are intended to generalize |  |
| Methods | | | |
| Case Vignettes | 4a | Describe the source of vignettes (e.g., medical education textbooks, patient records, patients’ descriptions, case studies, fictitious) |  |
|  | 4b | Report the sampling frame and rationale (e.g., which conditions, prevalence data, population-level statistics informed vignette selection or creation) |  |
|  | 4c | Report how statistical representativeness of vignettes was ensured (i.e., representative of prevalence within the sampling frame) |  |
|  | 4d | Report how content representativeness of vignettes was ensured (e.g., real cases, cases derived from patient records) |  |
|  | 4e | Report whether atypical cases were included or excluded |  |
|  | 4f | Specify the number of vignettes and provide a rationale (e.g., power analysis, feasibility) |  |
|  | 4g | Describe refinement and selection procedures (e.g., test-theoretical metrics such as item-total correlations or item difficulty indices) |  |
|  | 4h | State whether vignette content was lay-friendly or phrased for clinicians |  |
| Gold Standard Assignment | 5a | Describe how the reference standard was established |  |
|  | 5b | Report the number and background of experts involved |  |
|  | 5c | Explain how symptom checker outputs were mapped to triage categories |  |
| Pretest | 6 | State whether a pretest was conducted and provide a rationale |  |
| Tools | 7 | Specify which tools were evaluated and the exact version/date of access |  |
| Symptom Input Procedure | 8a | Specify the number and type of inputters (clinicians, laypeople, mixed) and whether they were sampled from the target population |  |
|  | 8b | Describe the entry instructions or provide them in an appendix |  |
|  | 8c | Explain how multiple inputters’ outputs were aggregated (e.g., majority vote) |  |
| Blinding | 9 | Report whether evaluators were blinded to the gold standard |  |
| Outcomes | 10a | Report which outcomes were assessed and why (e.g., overall accuracy, accuracy by triage level, safety, inclination to overtriage, comprehensiveness) |  |
|  | 10b | If multiple tools were tested: report relative performance metrics (e.g., Capability Comparison Score) |  |
|  | 10c | Report whether sensitivity analyses were conducted (e.g., different triage category mappings) |  |
| Data Analysis | 11a | Describe statistical methods used for data analysis |  |
|  | 11b | Report how missing data were handled (e.g., no outputs from symptom checkers) |  |
| Results | | | |
| Included Tools | 12 | Describe how many tools were identified, how many were tested by each person, how many provided advice, and how many were analyzed |  |
| Inputter Variability | 13 | Report inter-rater reliability across inputters |  |
| Tool Performance | 14 | Report performance of the included tools |  |
| Discussion | | | |
| Interpretation | 15 | Discuss results in the context of the intended use case, target population, and evaluation aim |  |
| User Study | 16 | State whether findings support progression to user-based evaluation, clinical trial, and/or whether users were already included in the evaluation |  |
| Limitations | 17 | Explain limitations (e.g., representativeness of vignettes, inputter variability) |  |
| Open Science | | | |
| Resources | 18a | State whether vignettes, protocols, and/or data are openly available and where they can be accessed |  |
|  | 18b | Report which open-source resources were used (e.g., vignettes from other authors, entry instructions provided by other authors, or software and packages such as symptomcheckR for analysis) |  |
| Funding and Conflicts of Interest | 19a | Report funding sources, their role in the study, and whether the developer of the tool provided financial or in-kind support |  |
|  | 19b | Declare any affiliations with developers of the evaluated tool(s) or other conflicts of interest |  |

Citation: Kopka, M. & Feufel, M.A. (2026). How to Evaluate the Accuracy of Symptom Checkers and Diagnostic Decision Support Systems: The Symptom Checker Accuracy Reporting Framework (SCARF). *JMIR Human Factors*. <http://dx.doi.org/10.2196/76168>

© 2025 Kopka & Feufel. This work is licensed under a Creative Commons Attribution 4.0 License, which allows unrestricted use, distribution, and reproduction in any format, provided the original work is properly cited.
