# Supplementary material for: How to Evaluate the Accuracy of Symptom Checkers and Diagnostic Decision Support Systems: Symptom Checker Accuracy Reporting Framework (SCARF)
Source: JMIR Hum Factors. 2026 Jan 16;13:e76168. doi: 10.2196/76168 (PMC12810947; doi:10.2196/76168)
Supplement: Multimedia Appendix 2 [file humanfactors-v13-e76168-s002.pdf]

Symptom Checker Accuracy Reporting Framework (SCARF) Checklist

Topic	Item Number	Item Description	Page Number
**Title & Abstract**			
Title	1	Title should indicate that the study evaluates a symptom checker or diagnostic decision support system	
Abstract	2	Summary of evaluation objective, methods, results, and conclusions	
Introduction			
Background and Objectives	3a	State the intended use case of the symptom checker (e.g., self-triage, emergency care triage)	
	3b	Define the target population to which findings are intended to generalize	
Methods			
Case Vignettes	4a	Describe the source of vignettes (e.g., medical education textbooks, patient records, patients' descriptions, case studies, fictitious)	
	4b	Report the sampling frame and rationale (e.g., which conditions, prevalence data, population-level statistics informed vignette selection or creation)	
	4c	Report how statistical representativeness of vignettes was ensured (i.e., representative of prevalence within the sampling frame)	
	4d	Report how content representativeness of vignettes was ensured (e.g., real cases, cases derived from patient records)	
	4e	Report whether atypical cases were included or excluded	
	4f	Specify the number of vignettes and provide a rationale (e.g., power analysis, feasibility)	
	4g	Describe refinement and selection procedures (e.g., test-theoretical metrics such as item-total correlations or item difficulty indices)	
	4h	State whether vignette content was lay-friendly or phrased for clinicians	
Gold Standard Assignment	5a	Describe how the reference standard was established	
	5b	Report the number and background of experts involved	
	5c	Explain how symptom checker outputs were mapped to triage categories	

Pretest	6	State whether a pretest was conducted and provide a rationale	
Tools	7	Specify which tools were evaluated and the exact version/date of access	
Symptom Input Procedure	8a	Specify the number and type of inputters (clinicians, laypeople, mixed) and whether they were sampled from the target population	
	8b	Describe the entry instructions or provide them in an appendix	
	8c	Explain how multiple inputters' outputs were aggregated (e.g., majority vote)	
Blinding	9	Report whether evaluators were blinded to the gold standard	
Outcomes	10a	Report which outcomes were assessed and why (e.g., overall accuracy, accuracy by triage level, safety, inclination to overtriage, comprehensiveness)	
	10b	If multiple tools were tested: report relative performance metrics (e.g., Capability Comparison Score)	
	10c	Report whether sensitivity analyses were conducted (e.g., different triage category mappings)	
Data Analysis	11a	Describe statistical methods used for data analysis	
	11b	Report how missing data were handled (e.g., no outputs from symptom checkers)	
**Results**			
Included Tools	12	Describe how many tools were identified, how many were tested by each person, how many provided advice, and how many were analyzed	
Inputter Variability	13	Report inter-rater reliability across inputters	
Tool Performance	14	Report performance of the included tools	
**Discussion**			
Interpretation	15	Discuss results in the context of the intended use case, target population, and evaluation aim	
User Study	16	State whether findings support progression to user-based evaluation, clinical trial, and/or whether users were already included in the evaluation	
Limitations	17	Explain limitations (e.g., representativeness of vignettes, inputter variability)	
**Open Science**			
Resources	18a	State whether vignettes, protocols, and/or data are openly available and where they can be accessed	

	18b	Report which open-source resources were used (e.g., vignettes from other authors, entry instructions provided by other authors, or software and packages such as symptomcheckR for analysis)	
Funding and Conflicts of Interest	19a	Report funding sources, their role in the study, and whether the developer of the tool provided financial or in-kind support	
	19b	Declare any affiliations with developers of the evaluated tool(s) or other conflicts of interest	

Citation: Kopka, M. & Feufel, M.A. (2026). How to Evaluate the Accuracy of Symptom Checkers and Diagnostic Decision Support Systems: The Symptom Checker Accuracy Reporting Framework (SCARF). *JMIR Human Factors*. http://dx.doi.org/10.2196/76168

© 2025 Kopka & Feufel. This work is licensed under a Creative Commons Attribution 4.0 License, which allows unrestricted use, distribution, and reproduction in any format, provided the original work is properly cited.
